# Agrarian diet and diseases of affluence – Do evolutionary novel dietary lectins cause leptin resistance?

**DOI:** 10.1186/1472-6823-5-10

**Published:** 2005-12-10

**Authors:** Tommy Jönsson, Stefan Olsson, Bo Ahrén, Thorkild C Bøg-Hansen, Anita Dole, Staffan Lindeberg

**Affiliations:** 1 Department of Clinical Sciences, Lund University, Lund, Sweden; 2 Department of Ecology, The Royal Veterinary and Agricultural University, Copenhagen, Denmark; 3 Institute of Molecular Pathology, University of Copenhagen, Copenhagen, Denmark

## Abstract

**Background:**

The global pattern of varying prevalence of diseases of affluence, such as obesity, cardiovascular disease and diabetes, suggests that some environmental factor specific to agrarian societies could initiate these diseases.

**Presentation of the hypothesis:**

We propose that a cereal-based diet could be such an environmental factor. Through previous studies in archaeology and molecular evolution we conclude that humans and the human leptin system are not specifically adapted to a cereal-based diet, and that leptin resistance associated with diseases of affluence could be a sign of insufficient adaptation to such a diet. We further propose lectins as a cereal constituent with sufficient properties to cause leptin resistance, either through effects on metabolism central to the proper functions of the leptin system, and/or directly through binding to human leptin or human leptin receptor, thereby affecting the function.

**Testing the hypothesis:**

Dietary interventions should compare effects of agrarian and non-agrarian diets on incidence of diseases of affluence, related risk factors and leptin resistance. A non-significant (p = 0.10) increase of cardiovascular mortality was noted in patients advised to eat more whole-grain cereals. Our lab conducted a study on 24 domestic pigs in which a cereal-free hunter-gatherer diet promoted significantly higher insulin sensitivity, lower diastolic blood pressure and lower C-reactive protein as compared to a cereal-based swine feed. Testing should also evaluate the effects of grass lectins on the leptin system in vivo by diet interventions, and in vitro in various leptin and leptin receptor models. Our group currently conducts such studies.

**Implications of the hypothesis:**

If an agrarian diet initiates diseases of affluence it should be possible to identify the responsible constituents and modify or remove them so as to make an agrarian diet healthier.

## Background

In this paper we look at global variation in the prevalence of diseases of affluence [1], such as obesity, cardiovascular disease and diabetes type 2 [2,3], between agrarian and non-agrarian societies. This societal division refers to differences in staple foods. The diet of an agrarian society is based on large amount of seeds from grass such as cereals (e.g. wheat, rice, maize). Cereals are per definition rare or absent in a non-agrarian diet. Non-agrarian societies can be further divided into hunter-gatherer and horticultural societies. The diet of a hunter-gatherer society is based on hunting, fishing and gathering wild plants and insects. Hunting and gathering is thought to represent the original mode of life common to all prehistoric humans during the Palaeolithic (i.e. the Old Stone Age 2.6 million-10, 000 years ago) [4,5]. Horticultural societies obtain the bulk of their food from gardening, which sometimes implies heavy dependence on a single starchy cultivar such as a root crop (e.g. manioc).

### Global epidemiologic pattern

Among agrarian societies there is considerable variation both in time and place in the prevalence of diseases of affluence [6-8]. The cause behind the initiation and progression of diseases of affluence are most certainly multi-factorial and probably several factors need to be present to a sufficient degree for these diseases to appear clinically. Among agrarian societies some diseases of affluence, such as obesity and type 2 diabetes, are associated with increasing westernization and urbanization, although some less westernized countries such as China and countries in sub-Saharan Africa did have more cases of diabetes in rural than urban areas in 1995 [6,7]. Several risk factors for obesity and diabetes type 2, such as low physical activity and a sedentary lifestyle with prolonged TV watching, are thus associated with westernization and urbanization, which perhaps explain their association with these diseases [9]. However, for other diseases of affluence, such as stroke and CHD, some of the varying prevalence among agrarian societies is puzzling with no consistent association with westernization, urbanization or rise in risk factors [8,10]. Indeed, CHD was reportedly rare in developed populations until the early 1900s with major increases in the occurrence and mortality rate from the disease in the 1930s, but with as much as five-fold differences in CHD mortality rates between European countries such as Poland and Spain [8]. Some of these differences in time and place may be explained by variation in known risk factors [11,12]. A few agrarian societies, like the Amazon-dwelling Brazilian Indian tribe Amondava, reportedly lack diseases of affluence, which possibly is due to a recent and small shift in diet incorporating small amounts of cereals in an otherwise non-agrarian diet [13]. Such a pattern of differentially delayed onset of the various diseases of affluence has been described [2]. However, the global epidemiological pattern suggests that almost all agrarian societies have some prevalence of diseases of affluence. In contrast, diseases of affluence have been virtually absent among many non-agrarian societies in Melanesia, Malaysia, Africa, South America and the Arctic [2,5,14]. One such traditional population are the horticultural Trobriand Islanders with a mortality from atherothrombotic circulatory diseases which apparently is close to zero, even though they have access to abundant sources of food, smoke heavily and have a fair share of elderly people [2,14]. These disproportionate differences between agrarian and non-agrarian societies are even larger with regard to the incidence of non-infectious stroke [10,15]. Moreover, when people living in non-agrarian societies migrate to an agrarian society or when their own society becomes agrarian they contract diseases of affluence [2,14], which illustrates the general rule that there is no genetic protection against diseases of affluence, only genetic variation in degree of susceptibility [2].

The global epidemiological pattern of varying prevalence of diseases of affluence thus suggests that some environmental factors specific to agrarian societies could initiate these diseases. There are many such candidate environmental factors, and in this paper we study cereals, the clearest defining dietary difference between an agrarian and non-agrarian diet. Since nothing in biology makes sense except in the light of evolution [16], we look at the cereal component of human diet from an evolutionary perspective.

### Human diet and evolution

The grasses emerged between 65 and 55 million years ago [17]. Since the last common ancestor of living primates, including humans, emerged before this time, some 90 to 65 million years ago, it cannot have had a diet consisting of seeds from grass [18,19]. Subsequent evolution of our primate ancestors up until 4–8 million years ago is thought to have taken place in the trees [20-23], where almost all potential plant food comes from dicotyledonous species [24] and the monocotyledonous grasses are absent [17]. The archaeological evidence during the last four million years of evolution towards *Homo sapiens *suggests that if grass seeds were being incorporated into the diet of our ancestors, they probably only contributed a small part [25]. *Homo sapiens *emerged about 200,000 years ago [26,27], and seeds from grass were probably not key dietary staples of *Homo sapiens *hunter-gatherers [28]. About 10,000 years ago (e.g. 500 generations) some populations invented agriculture [28], and their descendents possibly have some genetic adaptation to an agrarian diet such as lower prevalence of celiac disease and related HLA genotypes [29,30]. However, many populations shifted to agrarian diet more recently, between 1–100 generations ago, which from an evolutionary perspective is a very short time to admit any measurable sign of adaptation [31]. Thus, when examining human diet from an evolutionary perspective, it makes sense that humans with an evolutionary novel agrarian diet could suffer from diseases of affluence due to insufficient adaptation [31]. Many metabolic factors and pathways are important in the onset and development of diseases of affluence. When looking for metabolic signs of such insufficient adaptation, one of the more relevant associations is that between diseases of affluence and leptin resistance, an acquired insensitivity to high levels of leptin [32-38].

### Leptin resistance

Leptin acts as a signal to the brain to inhibit food intake and enable the storage in adipocytes of surplus calories while simultaneously protecting peripheral non-adipose tissue from toxic effects of intracellular lipid overload [39]. Leptin also affects the growth of blood vessels and bone; the immune system; glucose- and fat metabolism and the reproductive system [32,40]. Leptin administered peripherally in animal models such as rodents promotes weight loss and satiation, but peripheral administration of leptin in obese human does not promote significant weight loss [32,41]. This difference in effect together with the observation that most obese humans have high levels of leptin suggest that leptin resistance causes human obesity [32,41]. Sometimes end-organ resistance can be caused by mutations in hormone receptors, which has been described for several hormones. The pathophysiology of acquired forms of end-organ resistance to hormones such as insulin and leptin has been elusive [42]. The differing results from leptin administration implies that the detailed actions of leptin in energy metabolism are different in humans versus experimental animals such as rodents [43]. This difference could be genetically based and possibly an adaptation of the human or experimental animal leptin system to some environmental factors affecting their respective ancient ancestors. But this difference could also be due to an insufficient adaptation to some environmental factors, which are now affecting the leptin system of humans or experimental animals. To address these different possibilities we turn to recent studies on the molecular evolution of the leptin gene.

### Molecular evolution of leptin

The hominoids (gibbon, orang-utan, gorilla, chimpanzee, early human and modern human) emerged 25–30 million years ago [19]. Studies on the molecular evolution of leptin have shown a significant increase of non-synonymous to synonymous changes [31] in the ancestral line of primates giving rise to hominoids, and this significant increase is also relative to descendant hominoid species such as humans [43]. This implies that the ancestral line of primates giving rise to hominoids probably acquired several positive non-synonymous changes of their leptin gene due to adaptations, and that the leptin genes of humans have not changed much since the emergence of hominoids. Thus, based on findings from previous section on human diet and evolution, it is very unlikely that human leptin could be specifically adapted to an agrarian diet. Furthermore, similar studies on molecular evolution have shown high similarity of leptin genes in such diverse species as mouse, rat, chicken and turkey, which was ascribed to convergent or parallel evolution [44]. Since many mammals, which share the same distant common ancestor with these species, do not have similar genes, it seems plausible that this high similarity is due to convergent evolution and not parallel evolution [31]. This implies that natural selection has caused the leptin genes of these bird and rodent species to be highly similar by adapting them to some similar factor(s) in the environment of their ancient ancestors. Diet is an important environmental factor, as exemplified by primates, where it affects basal metabolic rate, size, reproduction and locomotion [19]. Since leptin is a regulator of appetite, energy metabolism and reproduction it could well be subject to forces of natural selection due to diet. Except for a diet containing seeds from grass, it is hard to discern an environmental characteristic shared by diverse rodent and bird species which is sufficient to explain such high similarity of leptin genes [45-47]. Thus, it is possible that leptin of these rodent and bird species are specifically adapted to a diet including large amounts of seeds from grass. It follows that such a diet possibly imposes problems to the human leptin system, which we have concluded is not specifically adapted to such a diet. The studies on molecular evolution of leptin thus indicate that the differing results from leptin administration in humans and experimental animals could be due to adaptation of mouse and rat leptin and insufficient adaptation of human leptin to a diet including large amounts of seeds from grass. When looking for constituents of seeds from grass explaining these differences, we find the properties of lectins interesting.

### Lectins

Lectins are proteins abundant in the virus, bacteria, animal and plant kingdom, which bind reversibly to specific sugar structures (for most references and background see [48,49]). Different classes of plants, such as mono- and dicotyledonous, have different classes of lectins with differing biochemical properties, and there is a subclass of lectins only found in grasses like cereals. Many plant lectins are thought to play a role in the plants defence against being eaten. Accordingly, plant lectins have an obvious preference for binding to sugar structures of animal, fungal or microbial origin, and are usually at highest concentrations in plant parts essential for reproductive success such as seed germs. The intensively studied lectin wheat germ agglutinin (WGA), which protects against insects and fungi [49], is present in wheat seed in both the germ and the gluten part of endosperm [50]. Peptides behaving in a lectin-like manner have also been obtained upon cleavage of gliadin in gluten [51]. Sourdough lactic acid bacteria hydrolyse gliadin peptides and inhibit their lectin-like behaviour [52], which perhaps explains some of the unexplained health effects of probiotics [53]. White flour consumed by humans contains a high proportion of gluten and has agglutinating activity suggestive of lectins [54-57]. Thus, lectins are present in our food, they are heat-stable and resistant to breakdown in the gastrointestinal tract, they bind to the surface epithelium of the digestive tract and they can lead to anti-nutritional, mild allergic or other subclinical effects in humans and animals [48,49]. Lectins can also be transported through the gut wall into the blood circulation, where they directly influence peripheral tissues and body metabolism through the binding to glycosylated structures, such as the insulin receptor, the epidermal growth factor receptor and the interleukin 2 receptor [57-65]. WGA have effects on activation of the epidermal growth factor receptor [61], mitogenesis [66], agglutination of red blood cells [48], activation of platelets and cell adhesion molecules [67] and vascular permeability [68-70]. WGA also have several effects related to autoimmunity, allergy and inflammation [57,71]. WGA binds to several types of mammalian cells including pancreatic duct epithelial cells [72], prostatic cancer cells [73], arterial macrophages and smooth muscle cells [74,75], glomerular capillary walls, mesangial cells and tubules of human kidney [59]. Human serum contains antibodies against WGA and lectins of soybean and peanut [76]. Hence, lectins have sufficient properties to affect the leptin system indirectly, through effects on metabolism central to the proper function of the leptin system, and possibly also directly through interaction with leptin or the leptin receptor. The intriguing possibility of a direct interaction between lectin and the leptin system is worth some additional comments.

### Possible direct interaction between lectin and the leptin system

The studies on molecular evolution of leptin indicated adaptation of rodent leptin and insufficient adaptation of human leptin to a diet including large amounts of seeds from grass. This adaptation and lack thereof could also involve the leptin receptor, since leptin and leptin receptor coevolves due to interdependency for signalling. An adaptation of the leptin gene could thus be to avoid disturbed function of either leptin or the leptin receptor. It would be interesting to see results from studies on molecular evolution of the leptin receptor, but such studies are unfortunately lacking. However, when considering direct lectin interaction with leptin or the leptin receptor, this interaction could be with either or with both. Lectins binding to sugar structures of a membrane receptor can mimic or block the effect of the physiological ligand [48,61,62,65,77-82]. Leptin is not glycosylated, but the leptin receptor is and lectins binding to different leptin receptor glycosylations might explain different leptin binding affinity [83,84], as observed by Livingston and Purvis in their study on WGA and the insulin receptor [63]. Thus, dietary lectins could possibly bind to the leptin receptor and affect its function, which could translate into diseases of affluence as indicated by studies on effects of single nucleotide polymorphisms on the function of leptin and the leptin receptor [85-91].

## Presentation of the hypothesis

The global pattern of varying prevalence of diseases of affluence suggests that some environmental factor specific to agrarian societies could initiate these diseases [2,14]. We propose that cereals, the clearest defining dietary difference between an agrarian and non-agrarian diet, could be such an environmental factor. Through previous studies in archaeology [17-25,28,31] and molecular evolution [43,44] we conclude that humans and human leptin system are not specifically adapted to a cereal-based diet, and that leptin resistance associated with diseases of affluence [32-38] could indicate insufficient adaptation to such a diet. As for the constituent(s) of cereals causing leptin resistance as a sign of insufficient adaptation, we propose lectins as a candidate with sufficient properties. Cereal lectins are specific to cereals [48,49], they are present in our food [50,51,54-57], they enter our systemic circulation and have many reported effects in our body including the binding to receptors, such as the insulin receptor, the epidermal growth factor receptor and the interleukin 2 receptor [48,57-75]. Cereal lectins could thus cause leptin resistance either indirectly, through effects on metabolism central to the proper functions of the leptin system, and/or directly, through binding to human leptin or leptin receptor, thereby affecting the function. The intriguing possibility of direct interaction between lectin and the leptin receptor could alter the function of the leptin receptor and translate into diseases of affluence [48,61-63,65,77-82,84-91].

## Testing the hypothesis

The hypothesis that an agrarian diet could initiate diseases of affluence should ideally be tested in prospective diet interventions comparing this diet with non-agrarian diets. Hard end-points should be various diseases of affluence and soft end-points should be their respective risk factors, specifically including leptin resistance. The only relevant human controlled intervention trial with hard end-points that we are aware of found a non-significant (p = 0.10) increase of cardiovascular mortality in CHD patients who were advised to eat more whole-grain cereals compared to those who were not advised to eat more whole-grain cereals [92]. We performed a trial on 24 domestic pigs in which a cereal-free hunter-gatherer diet promoted significantly higher insulin sensitivity, lower diastolic blood pressure and lower C-reactive protein as compared to a cereal-based swine feed (submitted). A prospective observational study on intake of refined grains as part of a "western diet" pattern showed a positive association with increased risk for type 2 diabetes [93]. Although the foods with major contributions to the "western diet" pattern were all positively associated with increased risk for type 2 diabetes, the consumption of refined grains remained significantly associated with the risk for type 2 diabetes when the foods with major contributions were modelled simultaneously [93]. However, the same study also showed a reduced risk for type 2 diabetes with a high intake of whole grain as part of a "prudent" diet pattern [93], and whole grains are reportedly also inversely related to weight gain, even after multivariate analysis for several indicators of a healthy living such as non-smoking and physical activity [94]. Accordingly, there are contradictory results from studies on effects of cereal grains on diseases of affluence. If not due to confounding factors, this is possibly explained by beneficial effects of whole grains as compared to refined grains, including higher fiber and micronutrient content, coupled with the usually inverse relationship between intake of whole and refined grain [94].

Evaluating the effects of grass lectins on the leptin system in vivo by diet interventions or in vitro in various leptin and/or leptin receptor models could test the hypothesis that cereal lectins might be the cause of leptin resistance. Our group currently conducts such studies. If dietary lectins could inhibit leptin binding and cause leptin resistance, then the proportion of leptin bound to the soluble leptin receptor in plasma should be lower in more leptin resistant humans on an agrarian diet, and this proportion should also increase with lower intake of dietary lectins. This is supported by the observations that the proportion of leptin bound to the soluble leptin receptor in plasma is lower in supposedly leptin resistant obese humans [95], and that this proportion increases after fasting in obese but not in lean humans [96]. The fasting state is obviously not an ideal situation for a direct comparison of the different effects of agrarian and non-agrarian diets, but in the absence of such studies fasting should cause less agrarian lectins to inhibit leptin binding. Further support comes from earlier studies from our laboratory on leptin levels in populations at a transitional stage from gathering to agricultural systems [97,98]. In addition, the recent finding that total leptin and free leptin both correlate with the dietary carbohydrate content, whereas bound leptin is associated with resting energy expenditure [99], seem to support our hypothesis.

## Implications of the hypothesis

If an agrarian diet initiates diseases of affluence it should be possible to identify the responsible constituents and modify or remove them so as to make the agrarian diet healthier. Furthermore, in animal experiments, the possible species-specific differences in adaptation to diets outlined in this article and their effects on studied parameters should be kept in mind when choosing the animal and the animal feed for the study. Furthermore, if cereal lectins should appear to have significant effects on human metabolism, then it is suggested that other plant lectins like peanut-lectin should be investigated in this regard as well.

## Competing interests

The author(s) declare that they have no competing interests.

## Authors' contributions

TJ conceived of and wrote the article. SO and SL conceived of and participated in the design of the article, and revised it critically for important intellectual content. BA, TB and AD have been involved in drafting the manuscript and revising it critically for important intellectual content. All authors read and approved the final manuscript.

## Pre-publication history

The pre-publication history for this paper can be accessed here:


